# Supplementary material for: T2-High Endotype and Response to Biological Treatments in Patients with Bronchiectasis
Source: Biomedicines. 2021 Jul 2;9(7):772. doi: 10.3390/biomedicines9070772 (PMC8301446; doi:10.3390/biomedicines9070772)
Supplement: Supplementary file 1 [file biomedicines-09-00772-s001.zip › biomedicines-1248287-supplementary.pdf]

biomedicines

MDPI

Online supplement for

T2-High Endotype and Response to Biological Treatments in Patients with *Bronchiectasis*

Martina Oriano [1,2], Andrea Gramegna [1,2], Francesco Amati [3,4], Alice D'Adda [1], Michele Gaffuri [5,6], Marco Contoli [7], Francesco Bindo [1,2], Edoardo Simonetta [1,2], Carlotta Di Francesco [1,2], Martina Santambrogio [1], Giovanni Sotgiu [8], Francesco Blasi [1,2] and Stefano Aliberti [3,4,*]

1 IRCCS, Ca' Granda Ospedale Maggiore Policlinico, Respiratory Unit and Cystic Fibrosis Adult Center, 20122 Milan, Italy; martina.oriano@unimi.it (M.O.); andrea.gramegna@unimi.it (A.G.); alice.dadda@policlinico.mi.it (A.D.); francesco.bindo@unimi.it (F.B.); edoardo.simonetta@unimi.it (E.S.); carlotta.difrancesco@unimi.it (C.D.F.); msantambrogio@gmail.com (M.S.); francesco.blasi@unimi.it (F.B.)
2 Department of Pathophysiology and Transplantation, Università degli Studi di Milano, 20122 Milan, Italy
3 Department of Biomedical Sciences, Humanitas University, Pieve Emanuele, 20090 Milan, Italy; francesco.aamati@gmail.com
4 IRCCS, Humanitas Research Hospital, Respiratory Unit, Via Manzoni 56, Rozzano, 20089 Milan, Italy
5 Fondazione IRCCS, Ca' Granda Ospedale Maggiore Policlinico, Department of Otolaryngology and Head and Neck Surgery, 20122 Milan, Italy; michele.gaffuri@policlinico.mi.it
6 Department of Clinical Sciences and Community Health, University of Milan, 20122 Milan, Italy
7 Respiratory Unit, Department of Translational Medicine, University of Ferrara, 44121 Ferrara, Italy; ctm@unife.it
8 Clinical Epidemiology and Medical Statistics Unit, Department of Medical, Surgical and Experimental Sciences, University of Sassari, 07100 Sassari, Italy; gsotgiu@uniss.it
* Correspondence: stefano.aliberti@hunimed.eu

Table S1. Clinical characteristics of the cohort and of the cohort without patients with concomitant asthma.

Variable		Total Population (*n* = 249)	Pure Bronchiectasis Without Asthma (*n* = 203)
**Demographics**			
Sex (female) , *n* (%)		193 (77.5%)	160 (78.8%)
Age, median (IQR) years		63.0 (50.0, 71.0)	63.0 (51.0, 71.0)
Former/active smoker, *n* (%)		109 (43.8%)	91 (44.8%)
Body mass index [Kg/m2], median (IQR)		21.2 (19.2, 24.0)	21.2 (19.0, 24.0)
**Radiology**			
Reiff score, median (IQR)		4.0 (3.0, 6.0)	4.0 (3.0, 6.0)
**Disease severity**			
BSI, median (IQR)		5.0 (3.0, 8.0)	5.0 (3.0, 9.0)
BSI risk class, *n* (%)	Mild	87 (36.1%)	72 (36.7%)
	Moderate	95 (39.4%)	73 (37.2%)
	Severe	59 (24.5%)	51 (26.0%)
**Etiology**			
Idiopathic, *n* (%)		156 (62.7%)	125 (61.6%)
Primary immunodeficiency, *n* (%)		23 (9.2%)	19 (9.4%)
Post Infective, *n* (%)		22 (8.8%)	19 (9.4%)
Primary Ciliary Diskynesia, *n* (%)		10 (4.0%)	9 (4.4%)
Secondary Immunodeficiency, *n* (%)		10 (4.0%)	9 (4.4%)
ABPA, *n* (%)		3 (1.2%)	2 (1.0%)

Other* , *n* (%)		27 (10.8%)	20 (9.8%)
**Comorbidities**			
BACI, median (IQR)		0.0 (0.0, 3.0)	0.0 (0.0, 0.0)
Gastroesophageal reflux disease, *n* (%)		127 (51.0%)	102 (50.2%)
Cardiovascular diseases, *n* (%)		88 (35.3%)	75 (36.9%)
Rinosinusithis, *n* (%)		92 (36.9%)	65 (32.0%)
Osteoporosis, *n* (%)		44 (17.7%)	38 (18.7%)
Neoplastic disease, *n* (%)		33 (13.3%)	31 (15.3%)
Depression, *n* (%)		23 (9.2%)	19 (9.4%)
**Clinical status**			
Exacerbations previous year, median (IQR)		1.0 (0.0, 2.0)	1.0 (0.0, 2.0)
3+ exacerbations in the previous year, *n* (%)		37 (16.2%)	27 (14.4%)
>1 hospitalization in previous year, *n* (%)		23 (10.1%)	17 (9.0%)
mMRC, *n* (%)	0	150 (60.7%)	122 (60.4%)
	1	74 (30.0%)	59 (29.2%)
	2	11 (4.5%)	10 (5.0%)
	3	7 (2.8%)	7 (3.5%)
	4	5 (2.0%)	4 (2.0%)
	3-4	12 (4.9%)	11 (5.4%)
**Quality of life - QoL-B questionnaire**			
QoL-B questionnaire Physical, median (IQR)		66.7 (40.0, 80.0)	66.7 (40.0, 86.7)
QoL-B questionnaire Role, median (IQR)		66.7 (46.7, 86.7)	70.0 (53.3, 86.7)
QoL-B questionnaire Vitality, median (IQR)		55.6 (44.4, 66.7)	55.6 (44.4, 66.7)
QoL-B questionnaire Emotion, median (IQR)		75.0 (55.6, 91.7)	79.2 (58.3, 91.7)
QoL-B questionnaire Social, median (IQR)		75.0 (47.2, 83.3)	75.0 (50.0, 91.7)
QoL-B questionnaire Treatment Burden, median (IQR)		66.7 (55.6, 77.8)	66.7 (55.6, 77.8)
QoL-B questionnaire Health, median (IQR)		41.7 (25.0, 58.3)	41.7 (25.0, 58.3)
QoL-B questionnaire Respiration, median (IQR)		74.1 (59.3, 81.5)	74.1 (63.0, 85.2)
**Functional evaluation**			
FEV_1, median (IQR) %predict.		84.0 (70.8, 98.2)	84.0 (71.0, 99.2)
FEV_1 <50%predict. , median (IQR)		16 (6.7%)	13 (6.6%)
FEV_1 <35%predict. , median (IQR)		6 (2.5%)	6 (3.1%)
**Microbiology**			
Chronic infection, *n* (%)		83 (40.3%)	74 (43.3%)
Chronic infection *P. aeruginosa*, *n* (%)		45 (21.8%)	41 (24.0%)
Chronic Infection *H. Influenzae*, *n* (%)		16 (7.8%)	15 (8.8%)
Chronic Infection MSSA, *n* (%)		16 (7.8%)	14 (8.2%)
Chronic Infection MRSA, *n* (%)		4 (1.9%)	4 (2.3%)
Chronic Infection *S. maltophilia*, *n* (%)		4 (1.9%)	3 (1.8%)
Chronic Infection *Achromobacter*, *n* (%)		2 (1.0%)	2 (1.2%)
Chronic Infection *S. pneumoniae*, *n* (%)		1 (0.5%)	1 (0.6%)
Chronic Infection *A. fumigatus*, *n* (%)		2 (1.0%)	1 (0.6%)
Other chronic infection, *n* (%)		10 (4.9%)	7 (4.1%)
**Th2 high endotype**			
Blood eosinophils, median (IQR) cells·μL^{-1}		130.0 (80.0, 220.0)	120.0 (80.0, 200.0)
Oral FeNO, median (IQR) ppb		14.6 (8.5, 24.3)	14.6 (9.2, 21.9)

Definition of abbreviations: BSI= Bronchiectasis severity index; MRSA= Methicillin-Resistant Staphylococcus Aureus; MSSA= Methicillin- sensitive Staphylococcus Aureus; ABPA= Allergic bronchopulmonary aspergillosis. Data are presented as median (interquartile range) or *n* (%). * Connective Tissue Disease, Alpha1-antitrypsin deficiency, Rheumatoid arthritis, Ulcerative colitis, Aspiration, Asthma, COPD, Inflammatory bowel disease, NTM infection.

Table S2. Comparison of clinical characteristics of Th2 and non-Th2 high.

Variable		Th2 High Endotype (*n* = 88)	Non-Th2 High (*n* = 161)	*p*-Value
**Demographics**				
Sex (female), *n* (%)		61 (69.3%)	132 (82.0%)	0.022
Age, median (IQR) years		65.0 (54.8, 70.2)	62.0 (48.0, 71.0)	0.166
Former/Active smoker, *n* (%)		44 (50.0%)	65 (40.4%)	0.143
Body mass index [Kg/m^2], median (IQR) , *n* (%)		21.4 (19.0, 23.8)	21.2 (19.2, 24.0)	0.920
**Radiology**				
Reiff score, median (IQR)		4.0 (3.0, 6.0)	4.0 (2.0, 6.0)	0.116
**Disease severity**				
BSI, median (IQR)		6.0 (4.0, 10.0)	5.0 (3.0, 8.0)	0.071
**Etiology**				
Idiopathic, *n* (%)		57 (64.8%)	99 (61.5%)	0.341
Primary immunodeficiency, *n* (%)		6 (6.8%)	17 (10.6%)	
Post Infective, *n* (%)		4 (4.5%)	18 (11.2%)	
Primary Ciliary Dyskinesia, *n* (%)		2 (2.3%)	8 (5.0%)	
Secondary Immunodeficiency, *n* (%)		5 (5.7%)	5 (3.1%)	
ABPA, *n* (%)		2 (2.3%)	1 (0.6%)	
Other* , *n* (%)		12 (13.6%)	14 (8.8%)	
**Comorbidities**				
BACI, median (IQR)		0.0 (0.0, 3.0)	0.0 (0.0, 0.0)	0.003
Gastroesophageal reflux disease, *n* (%)		47 (53.4%)	80 (49.7%)	0.575
Cardiovascular diseases, *n* (%)		24 (41.4%)	55 (34.2%)	0.598
Rinosinusithis, *n* (%)		41 (46.6%)	51 (31.7%)	0.020
Osteoporosis, *n* (%)		18 (20.5%)	26 (16.1%)	0.395
Neoplastic disease, *n* (%)		12 (13.6%)	21 (13.0%)	0.895
Depression, *n* (%)		10 (11.4%)	13 (8.1%)	0.392
**Clinical status**				
Exacerbations previous year, median (IQR)		1.0 (0.0, 2.0)	1.0 (0.0, 2.0)	0.309
3 + exacerbations in the previous year, *n* (%)		17 (20.7%)	20 (13.7%)	0.167
> 1 hospitalization in the previous year, *n* (%)		11 (13.4%)	12 (8.2%)	0.211
mMRC, *n* (%)	0	46 (52.9%)	104 (65.0%)	0.267
	1	29 (33.3%)	45 (28.1%)	
	2	5 (5.7%)	6 (3.8%)	
	3	4 (4.6%)	3 (1.9%)	
	4	3 (3.4%)	2 (1.2%)	
	3-4	7 (8.0%)	5 (3.1%)	0.086
**Quality of life-QoL-B questionnaire**				
QoL-B questionnaire Physical, median (IQR)		63.4 (41.7, 80.0)	66.7 (40.0, 86.7)	0.455
QoL-B questionnaire Role, median (IQR)		66.7 (46.7, 86.7)	73.3 (51.6, 85.0)	0.726
QoL-B questionnaire Vitality, median (IQR)		55.6 (44.4, 77.8)	55.6 (33.3, 66.7)	0.279
QoL-B questionnaire Emotion, median (IQR)		79.2 (52.1, 100.0)	75.0 (57.0, 91.7)	0.726
QoL-B questionnaire Social, median (IQR)		58.3 (43.8, 83.3)	75.0 (50.0, 91.7)	0.199
QoL-B questionnaire Treatment Burden, median (IQR)		66.7 (66.7, 77.8)	66.7 (55.6, 77.8)	0.128
QoL-B questionnaire Health, median (IQR)		41.7 (19.4, 58.3)	41.7 (25.0, 58.3)	0.386
QoL-B questionnaire Respiration, median (IQR)		66.7 (54.9, 81.5)	77.8 (64.8, 85.2)	0.054
**Functional evaluation**				
FEV_1, median (IQR) %predict.		83.0 (66.0, 97.0)	86.0 (73.5, 100.5)	0.091
FEV_1 <50%predict. , *n* (%)		9 (11.1%)	7 (4.4%)	0.090

FEV_1 <35%predict. , *n* (%)	4 (5.0%)	2 (1.3%)	0.191
**Microbiology**			
Chronic infection, *n* (%)	26 (34.7%)	57 (43.5%)	0.213
Chronic infection *P. aeruginosa*, *n* (%)	16 (21.3%)	29 (22.1%)	0.893
Chronic Infection *H. Influenzae*, *n* (%)	3 (4.0%)	13 (10.0%)	0.123
Chronic Infection MSSA, *n* (%)	4 (5.3%)	12 (9.2%)	0.323
Chronic Infection MRSA, *n* (%)	1 (1.3%)	3 (2.3%)	0.632
Chronic Infection *S. maltophilia*, *n* (%)	2 (2.7%)	2 (1.5%)	0.568
Chronic Infection *Achromobacter*, *n* (%)	1 (1.3%)	1 (0.8%)	0.688
Chronic Infection *S. pneumoniae*, *n* (%)	0 (0.0%)	1 (0.8%)	0.448
Chronic Infection *A. fumigatus*, *n* (%)	2 (2.7%)	0 (0.0%)	0.06
Other chronic infection, *n* (%)	3 (4.0%)	7 (5.3%)	0.666

Definition of abbreviations: BSI= Bronchiectasis severity index; MRSA= Methicillin-Resistant Staphylococcus Aureus; MSSA= Methicillin- sensitive Staphylococcus Aureus; ABPA= Allergic bronchopulmonary aspergillosis. Data are presented as median (interquartile range) or *n* (%). * Connective Tissue Disease, Alpha1-antitrypsin deficiency, Rheumatoid arthritis, Ulcerative colitis, Aspiration, Asthma, COPD, Inflammatory bowel disease, NTM infection.
